# The Eye of the Common Octopus (*Octopus vulgaris*)

**DOI:** 10.3389/fphys.2019.01637

**Published:** 2020-01-14

**Authors:** Frederike D. Hanke, Almut Kelber

**Affiliations:** Lund Vision Group, Department of Biology, Lund University, Lund, Sweden

**Keywords:** vision, cephalopods, octopoda, visual function, optics

## Abstract

*Octopus vulgaris*, well-known from temperate waters of the Mediterranean Sea and a well-cited model species among the cephalopods, has large eyes with which it scans its environment actively and which allow the organism to discriminate objects easily. On cursory examination, the single-chambered eyes of octopus with their spherical lenses resemble vertebrate eyes. However there are also apparent differences. For example, the retina of the octopus is everted instead of inverted, and it is equipped with primary rhabdomeric photoreceptors rather than secondary ciliary variety found in the retina of the vertebrate eye. The eyes of octopus are well adapted to the habitat and lifestyle of the species; the pupil closes quickly as a response to sudden light stimuli mimicking a situation in which the octopus leaves its den in shallow water during daytime. Although the many general anatomical and physiological features of octopus vision have been described elsewhere, our review reveals that a lot of information is still missing. Investigations that remain to be undertaken include a detailed examination of the dioptric apparatus or the visual functions such as brightness discrimination as well as a conclusive test for a faculty analogous to, or in lieu of, color vision. For a better understanding of the octopus eye and the functions mediated by it, we suggest that future studies focus on knowledge gaps that we outline in the present review.

## Introduction

**Figure 1:**
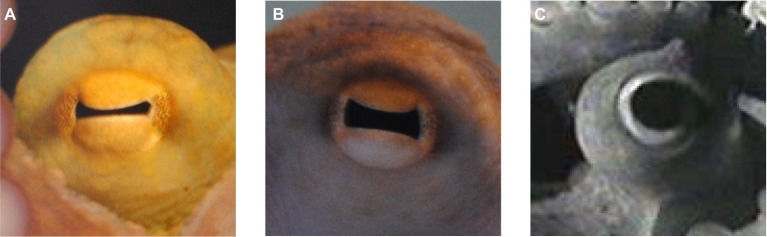
Pupil of *Octopus vulgaris*. **(A)** Constricted horizontal slit pupil in bright light, **(B)** intermediate pupil size, and **(C)** fully dilated pupil in dim light conditions.

If you have ever encountered an octopus, the way the animal looks at you is striking; you feel as if you are being scanned. The eyes are one of the prominent characteristics of the octopus but also of cephalopods in general. Already from outside, the eyes appear to be special. They are usually rather large with a diameter of approximately 20 mm (see section “Eye Size and Ocular Dimensions”), and their pupils often have conspicuous shapes (see Figure 1 and, for example, photos in Douglas, 2018). If one takes a closer look at eye morphology, the coleoid cephalopod eyes attract attention, as parallels can be drawn between the design of the camera type eyes of these molluscs and the design of vertebrate eyes, particularly those of fish (von Lenhossék, 1894; Packard, 1972). At the neuronal level, large parts of the cephalopod brain are dedicated to the processing of visual information as indicated by the size of their optic lobes (Young, 1960, 1971; Wells, 1966a; Maddock and Young, 1987).

In numerous studies on the eyes of many of the approximately 800 known species of cephalopods (Jereb and Roper, 2005, 2010; Jereb et al., 2014), we have learned a lot about specialized eye designs, for example, the pinhole eye of *Nautilus* (Hensen, 1865; Griffin, 1900; Wiley, 1902; Merton, 1905; Hurley et al., 1978; Muntz and Ray, 1984; Muntz, 1991; Barber, 2010), the asymmetrical eyes of *Histioteuthis* (Denton and Warren, 1968; Young, 1975; Wentworth and Muntz, 1989; Thomas et al., 2017) and the largest eyes on Earth, found in *Architeuthis* and *Mesonychoteuthis* (Nilsson et al., 2012), just to mention a few examples. In addition to reports regarding peculiar eye designs, researchers have worked on many aspects of the visual system in more common cephalopod genera such as *Octopus*, *Eledone*, *Sepia*, and *Loligo*. Young (1962a) pointed out basic similarities among the eyes of these genera, but at the same time, mentioned important differences between them. Because of these apparent differences, neither generalizing conclusions from one species to another, nor combining data from different species to derive overarching conclusions should be the method of choice.

This review aims to summarize the present knowledge regarding the eye and vision of a well-studied cephalopod, the common octopus, *Octopus vulgaris*. Thus, when we are referring to octopus in the text, data collected with *Octopus vulgaris* are considered; if data from other cephalopod species are included for comparison, the species name is indicated. We set out to collect information on vision in the common octopus as it is a prominent model species among cephalopods and has probably been the most-studied cephalopod species for more than 150 years. Especially in the mid-20th century, many studies were designed to unravel the discriminatory and cognitive abilities of this species using behavioral tests with visual stimuli (for example, see work by Boycott, Mackintosh, Messenger, Sutherland, Wells, and Young such as Boycott and Young, 1956; Young, 1956; Sutherland, 1957; Wells, 1960; Mackintosh, 1963; Messenger, 1968a). However, our understanding of vision in octopus is still patchy and has never been summarized specifically for this species. After a short, general introduction to *Octopus vulgaris* in general, it is the aim of the current review to gather, to the best of our knowledge, all information available on the eye of the common octopus. The collection of references can then form the basis for future investigations of vision and the visual faculties of this species. Accordingly, we will mention such future avenues in the text.

## General Introduction to *Octopus Vulgaris*

*Octopus vulgaris*, first described by Cuvier in 1797, belongs to the family *Octopodidae* encompassing more than 200 species. The genus *Octopus* constitutes a “catchall” genus (Jereb et al., 2014) for all species that possess two rows of suckers on the eight arms and an ink sac. The distribution of *Octopus vulgaris sensu stricto* (Jereb et al., 2014) covers the Mediterranean Sea, as well as the central and north-east Atlantic Ocean. The common octopus is said to be nocturnal (Woods, 1965; Altman, 1966; Kayes, 1974; Jereb et al., 2014), but it has been seen to shift its activity phase, for example in the presence of prey or predators (Meisel et al., 2013), and thus some studies report crepuscular or even diurnal activity (Mather, 1988; Meisel et al., 2003, 2006). In the presence of one of its many predators (Sanchez et al., 2015), the soft-shelled octopus either hides in dens, camouflages to the background with the help of a sophisticated system of pigment-filled chromatophores, electron-dense leucophores, and reflecting iridophores, or exhibits distinct behavioral displays (Packard and Sanders, 1971). The dens are inhabited only temporarily for a couple of days or weeks (Kayes, 1974; Mather and O’Dor, 1991). Octopus uses natural crevices or holes as hiding places or accumulates rocks and shells to build its own den. As a bottom feeder, foraging often seems to be tactile (Jereb et al., 2014), involving exploration of the surroundings with its arms, in search for crustaceans, fish, shelled molluscs or polychaetes (Mather, 1991; Boyle and Rodhouse, 2005; Mather et al., 2012; Sanchez et al., 2015). In addition, visual and chemical cues are most likely used to find prey (Boyle and Rodhouse, 2005). *Octopus vulgaris* is solitary, and the sexes only meet during mating (Hanlon and Messenger, 2018) when the male transfers spermatophore packages with its heterocotylus, an enlarged sucker on one of the arms, into the mantle cavity and oviduct of the female. At the end of the life cycle, the female lays 100,000–500,000 eggs bound together and glued to the ceiling of a den or to a rock. The female stays with the eggs for the duration of development, which can last up to 5 months, continuously caring for and defending the eggs. The female octopus does not feed during this period, digesting its own musculature in this last phase of its life (Jereb et al., 2014; Hanlon and Messenger, 2018). As a consequence, the female dies shortly after the eggs hatch. The 1–2 mm sized transparent hatchlings, called paralarvae, undergo a planktonic phase mostly in shallow (i.e., pelagic) waters that can last weeks to months before they settle on the sediment. The subsequent adult life stage can last up to 2 years during which octopus adopts a general benthic lifestyle but is still commonly found in pelagic waters. Specimens of *Octopus vulgaris* can reach a mantle length of up to 250 mm, a total length of over 1 m, and a body weight of more than 2 kg (Jereb et al., 2014).

Reader interested in the biology of cephalopods, including *Octopus vulgaris,* are referred to Hanlon and Messenger (2018), or to Jereb et al. (Jereb and Roper, 2005, 2010; Jereb et al., 2014, 2015).

## Eye Size and Ocular Dimensions

**Figure 2:**
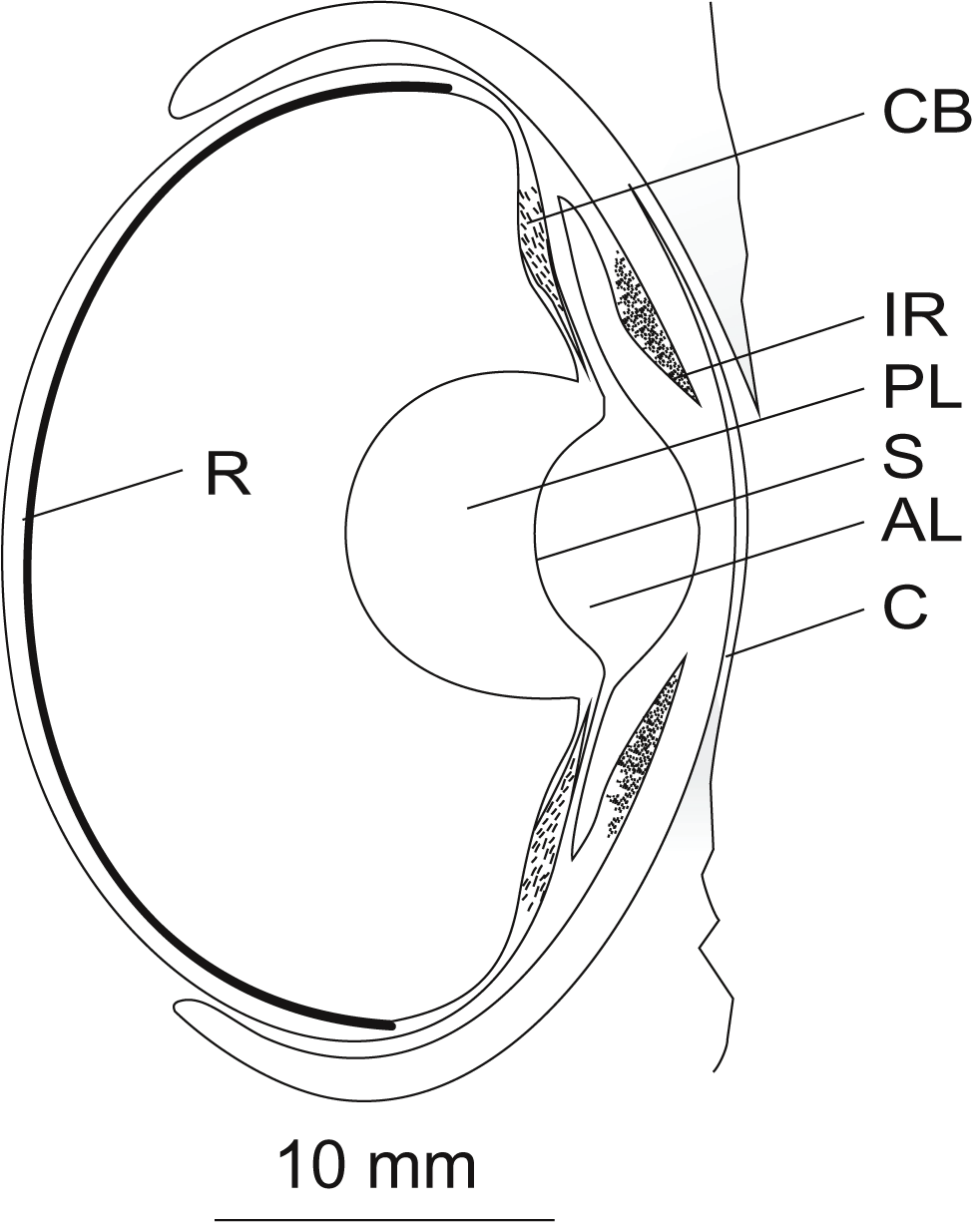
Schematic of the eye of *Octopus vulgaris* (longitudinal vertical section). Light falling on the eye of octopus first hits the cornea (C). Beyond the cornea, the light passes the anterior chamber and the pigmented mobile iris (IR) before it is refracted by the spherical lens. The lens, composed of an anterior (AL) and a posterior part (PL) separated by a septum (S), is suspended by the ciliary body (CB). Finally, the light hits the everted retina (R) in the back of the eye. A detailed description of the ocular structures is given in the text. The figure was adapted from frozen sections of an octopus eye and from Figure 1 in Budelmann (1994) and Figure 5 in (Wells, 1966b) displaying an eye of *Octopus* spec. Scale 10 mm.

Often, the eye of *Octopus vulgaris* (Figure 2) is described as large. In several studies, external eye dimensions are given. Beer (1897) measured an eye length (most likely axial eye length) of 17 mm in an octopus individual weighing 607 g. Additionally, Hanlon and Messenger (2018) documented an eye diameter of 20 mm in an individual weighing 205 g. Both values are within the range of eye diameters of 15–20 mm given by Fröhlich (1914a) for *Eledone moschata*, *Octopus macropus,* and *Octopus vulgaris*. According to Packard (1969), a really large octopus can have an eye with a diameter larger than 20 mm; however ‘really large’ is not further specified by this author. For comparison, the eyes of humans are, on average, 24 mm in diameter (Augusteyn et al., 2012). Given that adult humans weigh far more than an octopus, the octopus indeed has a large eye relative to body size/mass. The octopus eye is large even when compared to a nocturnal bird such as the tawny owl (*Strix aluco*), which weighs 400–800 g, and has an eye diameter of 23–29 mm (Brooke et al., 1999).

Beside external eye dimensions, no information on internal parameters such as ocular dimensions, radii of curvature, refractive indices or absorption coefficients of ocular media is available for octopus. These data would be required in order to develop detailed and informative optical models of the eye of octopus to further increase our understanding of how and what the octopus sees.

## Visual Fields and Eye Movements

The eyes of octopus are placed laterally and can be moved independently, with the eye axes occasionally deviating by up to 180 degrees (Heidermanns, 1928). To date, no measurements of visual field size are available for this species. From the eye placement of octopus, one could assume that octopus possesses a small binocular visual field, to the front and possibly to the back; however, Budelmann et al. (1997) dispute the existence of a binocular field in octopods. In any case, octopus certainly has large monocular visual fields, the space in which objects can be seen with one eye. This is consistent with the animals watching or tracking objects preferable with one eye (Heidermanns, 1928; Muntz, 1963; Byrne et al., 2002, 2004). The size of the monocular visual field is likely similar to that of *Sepia officinalis*. Model calculations in *Sepia* revealed that the visual field is limited by pupil size and that it is much smaller (Schaeffel et al., 1989) than the 177 degrees estimated by Messenger (1968b) for the horizontal plane.

The octopus can modify the space it can oversee by retracting and bulging out its eyes, or by rotational eye movements. The rotational eye movements that can turn the eye up to 80 degrees sideways in either direction (Budelmann and Young, 1984) are mediated by four oblique muscles that pass halfway around the eyeball. In total, each octopus eye has seven extra-ocular muscles, each innervated by a separate nerve (Glockauer, 1915; Budelmann and Young, 1984). In contrast, decapod cephalopods have up to 14 eye muscles that are innervated by only four nerves (Glockauer, 1915; Budelmann and Young, 1993).

Octopus also shows reflexive eye movements. When stimulated by a large field vertical grating rotating on an optokinetic drum, the animals perform compensatory eye, head, and body movements (Packard, 1969).

Future studies of the visual fields of octopus are highly desirable, particularly those that provide measurements of the putative binocular visual field and evaluate its implications for binocular depth perception, the monocular visual field, and the dynamic visual field, taking eye movements into account. Regarding eye movements, it remains to be determined whether the octopus can also turn its eyes upwards and downwards, and if so, to what degree.

## Eye Lid and Cornea

As is likely the case with all octopods, octopus possesses a ring-shaped muscular skin fold or bulge around the eye that can close in a manner comparable to an eye lid (von Lenhossék, 1894; Magnus, 1902). This eye lid-like structure closes over a cornea (Figure 2) which is hardly visible in the living octopus. This cornea has also been referred to as pseudo-cornea (Schöbl, 1877) or pseudo-corneal fold (Amoore et al., 1959). According to previous studies (Beer, 1897; Magnus, 1902), which are supported by our own observations, the cornea is not a component of the eye, meaning that the cornea cannot be extracted together with the underlying ocular structures. Moreover, it has a dorsal opening which brings the anterior chamber — the compartment between cornea and lens — in contact with the surrounding sea water (Amoore et al., 1959; Wells, 1966b); although this finding is not undisputed. As expected, the fluid within the anterior chamber has the same sodium concentration as seawater, however the potassium concentration has been found to be higher (Amoore et al., 1959).

A detailed analysis of the cornea is required to determine the functional role of the cornea in the eye of the octopus. Interesting insight in this often neglected structure could also be obtained by studying the histological fine structure of the cornea or its development during ontogeny.

## Pupil and Iris

One of the most prominent features of the octopus eye is its pupil (Figure 1). The cephalopod pupil is mobile, in contrast to the pupil of fishes, excluding the elasmobranchs (Douglas, 2018). The pupil of octopus is circular in darkness (Figure 1C), while in bright light, it constricts to a horizontal slit (Figures 1A,B) corresponding to the orientation of the central stripe of increased photoreceptor density on the retina (Muntz, 1977; and see section “Retina and Visual Function”). Compared to other cephalopods that can have U- or W-shaped pupils (e.g., cuttlefish), a slit-shaped pupil is a rather simple pupil design (Douglas, 2018).

In general, the octopus pupil adapts the eye to changes in ambient light. The advantage of the pupillary reaction is that it is faster than the alternative adaptation mechanisms which, in octopus, are pigment migration and the contraction/enlargement of the photoreceptors (Babuchin, 1864; Young, 1963). Pupil dynamics were recently examined in an octopus by Soto (2018). The individual studied, with a mantle length of approximately 6.5 cm, had a pupil area of 33 mm^2^ when the pupil was fully dilated. Pupil area decreased to approximately 4 mm^2^, or 12% of the dark-adapted pupil area, when the eye was exposed to bright light. Constriction of the octopus pupil was thus similar to or a little weaker than in other cephalopod species (Douglas et al., 2005; Bozzano et al., 2009; Matsui et al., 2016) such as *Sepia officinalis* or *Eledone cirrhosa* that constrict their pupils to 3% of the maximal area (Douglas et al., 2005). It took the octopus pupil 0.5–1.3 s to reach half maximum constriction defined as the t_50_ value. Most other cephalopod pupils examined so far also constricted quickly upon light exposure with t_50_ values ranging from 0.3 to 3 s (Douglas et al., 2005; McCormick and Cohen, 2012; Matsui et al., 2016). Thus, these pupils are adapted to fast light changes also occurring in the habitat of octopus, for instance when they are leaving the den in shallow water during daytime hours. In contrast, pupil constriction took 90 s in *Nautilus pompilius* (Hurley et al., 1978), a species that is most likely not experiencing drastic variations in ambient light in its habitat. The same probably holds true for *Japetella diaphana*, a deep sea octopus, whose pupil takes approximately 6 s to constrict (Douglas, 2018). In addition, the range of light intensities to which the pupil of *Octopus vulgaris* reacts with intermediate pupil sizes is narrow (Hess, 1905; Soto, 2018); the pupil already fully constricts in response to a luminance of approx. 20 cd/m^2^.

Axial light has a stronger effect on pupillary dilation than light from above (Soto, 2018), as described generally for cephalopods by Hess (1909, 1910) or McCormick and Cohen (2012). This “shadow effect” of the pupil for light from above might result in a more constant intensity of the retinal image than the illumination in the natural environment, in which most light is coming from above; this effect has so far only been described for *Sepia officinalis* (Mäthger et al., 2013).

Pupil dilation seems highly variable and individual (Magnus, 1902), and is also affected by factors other than ambient illumination (Weel and Thore, 1936). Octopus might constrict its pupil to camouflage the eye, allowing the animal to blend into the substrate, and the dilated pupil could serve as intra-specific deimatic signal, making the animal appear larger and more threatening to potential predators (Douglas, 2018).

Octopus does not show a consensual pupil response (Magnus, 1902; Weel and Thore, 1936). If only one eye is illuminated, only the pupil of this eye constricts, not the pupil of the non-illuminated eye. A non-consensual pupil response is adaptive in a species that has laterally placed eyes and watches objects predominantly with one eye (Heidermanns, 1928; Muntz, 1963; Byrne et al., 2002, 2004).

The octopus usually keeps the pupil horizontal, a reaction mediated by the statocysts that are required for the animal to maintain proper body and eye orientation (Boycott, 1960; Wells, 1960; Boycott et al., 1965). Only if the pupil is horizontal, and thus the orientation of the retinal receptors is fixed relative to the external world (see section “Retina and Visual Function”), the octopus is able to discriminate stimuli differing in orientation (Boycott and Young, 1956; Sutherland, 1957, 1963a; Wells, 1960; Young, 1960). This suggests that visual and proprioceptive input is not integrated in the brain.

The octopus pupil is bounded by the iris. According to Hess (1909), the cephalopod iris is not a structure of the inner eye but instead lies in form of a lobe in front/on top of the posterior chamber (Figure 2). The iris consists of five cell layers (Froesch, 1973): the external epithelium, a chromatophore and iridocyte layer, a layer of muscles and collagen strands, and the pigment epithelium. The chromatophores and the pigment epithelium absorb, while the iridophores reflect light, thereby changing the appearance of the eye, for instance when a threatened animal displays the dark eye bar over the eyes (Packard and Sanders, 1971). The muscles found in the iris are most likely sphincters, however, Froesch (1973) was unable to distinguish between sphincter and dilator. Brain regions and nerves involved in the pupillary reaction were described by Magnus (1902) as well as Weel and Thore (1936).

Soto (2018) described the pupillary reactions of only one octopus individual. It would be interesting to analyze more individuals to assess whether the data already obtained are representative for the species; in this case, the non-consensual pupil reaction could also be quantified. A future challenge might also be to further characterize the role of the pupil shape in modulating optical properties or for camouflaging the eye. Regarding the latter, an interesting study of pupil shape-mediated camouflage in skates was recently published (Youn et al., 2019).

## Lens and Accommodation

At first glance, the octopus lens, the main refracting structure within its eye, seems to be spherical (Figure 2). However, as the lens of *Octopus vulgaris* has not been measured, it might be slightly ellipsoidal, as is the case in other cephalopods (Sivak, 1982, 1991; Sroczynski and Muntz, 1985; Sivak et al., 1994). Fishes also have spherical lenses: however, in contrast to fish, the lens of octopus consists of an anterior and a posterior part divided by a septum (Figure 2; Budelmann, 1996). Each component is comprised of onion-like layers (Budelmann et al., 1997).

The lens develops from the lentigenic body, called “corpus epithelia” in early studies (Arnold, 1967). The cells of the lentigenic body are characterized by their larger size, prominent nuclei, intensely stained nucleoli, and cytoplasmic RNA. The lentigenic body lies in the front of the optic vesicle. Fine cytoplasmic processes of the lentigenic body form the lens primordium, which increases in size through the addition of further lentigenic processes to the surface (Arnold, 1967). Studies of the octopus lens have so far mainly focused on lens development and lens proteins (Arnold, 1967; Bon et al., 1967; Dohrn, 1970; Brahma, 1978) with the aim of understanding the convergent evolution of cephalopod and vertebrate lenses.

Beer (1897) examined accommodation in numerous cephalopod species including *Octopus vulgaris* and concluded that the octopus eye can, indeed, accommodate or adjust its focus. According to Beer, the octopus is myopic or short-sighted, in its resting state; thus its eyes are well-adapted to seeing objects nearby. Beer found that when the eye was electrically stimulated, refraction changed to a status close to emmetropia i.e., normal-sightedness. This change was not accompanied by a change in the curvature of the lens, but by a positional change as in fish (Land and Nilsson, 2002): the lens moved closer to the retina. The retraction of the lens was caused by the contraction of a ring-shaped muscle at the equator of the bulbus which is firmly associated with the ciliary body (Figure 2) that is a section of the uvea and serves to suspend the lens. Upon contraction, the ciliary body and lens are pulled against the retina. A prerequisite for these movements is that the eye bulbus of octopus is very soft and flexible.

Beer (1897) also assumed a myopic resting refractive state for *Sepia officinalis*. However, retinoscopic measurements in *Sepia officinalis* revealed emmetropia or slight hyperopia (Schaeffel et al., 1999). In the latter study, it was also speculated that the accommodation mechanism in *Sepia* involves the lens moving laterally, thus perpendicular relative to the pupillary axis of the eye. It is likely that new investigations of visual accommodation in octopus would also reveal a resting refractive state close to emmetropia. In general, octopus might not need elaborate accommodation abilities as its spherical lens with a short focal length, in conjunction with long receptor cells (see section “Retina and Visual Function”) most likely provide a large depth of focus (Budelmann et al., 1997).

There are a number of open questions related to the octopus lens, beginning first with the previously mentioned spherical shape of the lens. The second question relates to ocular transmittance. According to Denton and Warren (1968), octopus lenses should absorb ultraviolet (UV) light as octopus live close to the surface, whereas cephalopods living in the deep sea seem to have transparent lenses. However, this aspect needs to be studied in greater detail, as the statement by Denton and Warren (1968) is in contrast to a note by Hess (1910) in his work regarding the lenses of *Eledone* and *Sepia* which, according to his measurements, do not absorb light of any wavelength. As no details of the measurement procedure are given by Hess, we must assume that he was only able to measure in the visible part of the spectrum. Thus his note has to be treated with caution.

Third, very little is known about the optical properties of the lens of octopus. According to a side note in Sutherland (1963b), the lens is not astigmatic, thus the different meridians do not possess different refractive power. Most likely, it possesses a graded refractive index that compensates for longitudinal spherical aberration, such that axial and non-axial light rays are focused in the same focal plane, as in *Octopus pallidus* and *Octopus australis* (Jagger and Sands, 1999) or with some residual spherical aberration as in other cephalopod lenses (Sroczynski and Muntz, 1985, 1987; Sivak, 1991; Sivak et al., 1994; Kröger and Gislen, 2004; Sweeney et al., 2007); the lens of *Illex illecebrosus* seems to be overcorrected for spherical aberration (Sivak, 1982). In contrast to spherical aberration, the lenses of *Octopus* spec. do not seem to be corrected for chromatic aberration (Heidermanns, 1928; Jagger and Sands, 1999). In this regard, the nature of chromatic aberration — that is, a condition in which light of different wavelengths is focused differently — has to be re-evaluated in the context of color vision (see section “Visual Pigment and Color Vision”).

Finally, regarding the development of the split cephalopod lens, it is still unknown how the growth of the two components is coordinated. This question was already posed by Jacob and Duncan (1981) in the case of *Sepiola atlantica*, in which the anterior and posterior part of the lens are not closely electrically coupled. These authors also suggested studying whether the anterior and posterior halves of the lens are built from the same lens proteins.

## Retina and Visual Function

Although the eyes of vertebrates and coleoid cephalopods are similar in many aspects (Packard, 1972), the retinal designs of these two animal groups differ drastically. Cephalopods have everted retinae with the rhabdomeric photoreceptors pointing towards the light (Fröhlich, 1914a) in contrast to the inverted retinae with ciliary photoreceptors in vertebrates. Moreover, in contrast to the multilayered vertebrate retinae, cephalopod retinae mainly contain the photoreceptors. Cephalopod photoreceptors are primary receptor cells, each with its own axon, whereas the vertebrate photoreceptors are secondary receptor cells derived from epithelial cells. The axons of octopus photoreceptors project directly to the large optic lobes, where the visual information is processed (Young, 1960, 1971; Wells, 1966a; Maddock and Young, 1987). In vertebrates, the processing of the visual information already begins in the inner retina, before visual signals pass into the brain *via* the optic nerve.

**Figure 3:**
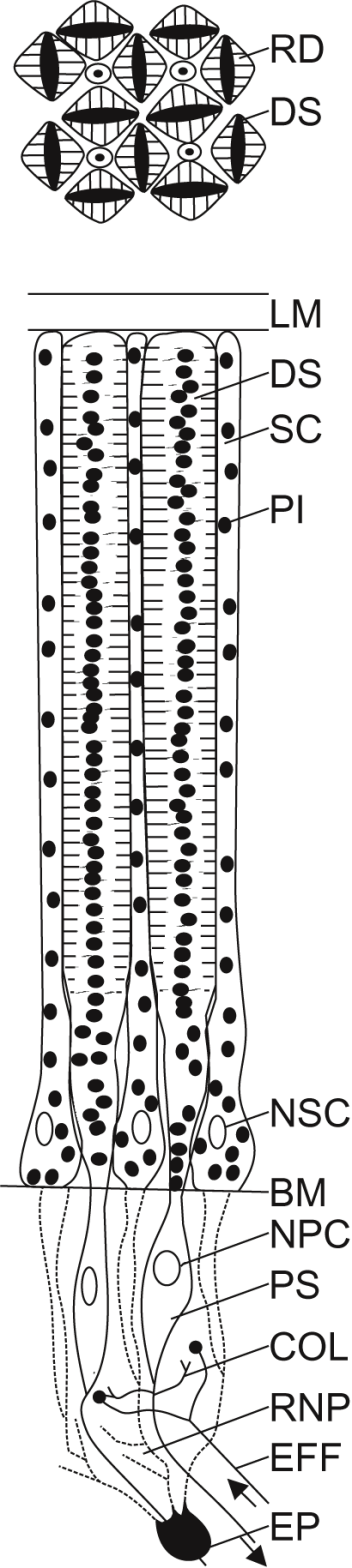
Schematic diagram of the retina of *Octopus vulgaris*. A limiting membrane (LM) shields the retina towards the posterior chamber. In the distal part of the retina are found the distal segments (DS) of the photoreceptors and supporting cells (SC). Pigment granules (PI) can be found within the photoreceptors and the supporting cells. The cross section through the distal retina (upper diagram), shows the regular arrangement of the distal segments of the photoreceptors (DS) that possess two rhabdomeres (RD) each, facing opposite sides of the cell, and separated by pigment (PI). Four rhabdomeres from four neighboring receptors form a rhabdom. While the nuclei of the supporting cells (NSC) are situated in the distal retina, the nuclei of the photoreceptor cells (NPC) are found in their proximal segments (PS) in the proximal retina, beyond the basal membrane (BM). In the proximal retina, within the retinal nerve plexus (RNP), photoreceptors are interconnected by collateral fibers (COL) from the proximal segments of the photoreceptors, and photoreceptors interact with efferents (EFF) from the optic lobe. Epithelial cells (EP), considered to be retinal glia cells, seem to form processes (dashed lines) that lie between the inner segments of the retinal cells. The schematic diagram of the octopus retina was adapted from previously published drawings (Babuchin, 1864; Schultze, 1867; Grenacher, 1884; Wolken, 1958; Moody and Parriss, 1960, 1961; Young, 1960, 1962b, 1971; Boycott et al., 1965; Yamamoto et al., 1965).

We will now describe the retina of *Octopus vulgaris* in detail (Figure 3). A limiting membrane shields the retina towards the posterior chamber. The limiting membrane might be a secretion of the supporting cells (von Lenhossék, 1894) that lie between the rhabdoms in the distal retina; there are about as many supporting cells as rhabdoms (Young, 1963).

The retina itself is densely packed with photoreceptors; their density is highest in a central horizontal stripe (Young, 1960, 1962b, 1963, 1971). At its distal end, oriented towards the light, each photoreceptor carries two rhabdomeres facing opposite sides. Four rhabdomeres belonging to four photoreceptors form a square rhabdom (Figure 3), which is analogous to the rhabdom of arthropods. The square arrangement of the rhabdoms is very regular, although there are also some cells which are particularly small that are not organized in arrangements of four (Young, 1963). Despite this very regular receptor arrangement, as well as corresponding regular distributions of the dendrites in the plexiform layer in the optic lobe (Young, 1960), the octopus is only able to discriminate stimuli differing in orientation (Boycott and Young, 1956; Sutherland, 1957, 1963a; Wells, 1960; Young, 1960) when the eye is oriented such that the pupil is horizontal, that is when the statocysts are functioning normally (Boycott, 1960; Wells, 1960; Boycott et al., 1965). The regular receptor arrangement plays an important role for the polarization sensitivity of the eye of octopus (see section “Dichroism of the Retina and Polarization Sensitivity”).

The two rhabdomeres of each photoreceptor are separated by screening pigment in the cell body (Figure 3). Additional pigment is found in the processes of the supporting cells between the distal segments of the photoreceptors. The migration of this screening pigment to the bases/tips of the photoreceptor and perhaps also the supporting cells (Babuchin, 1864; Young, 1963), in combination with enlargement/contraction of the photoreceptors and the constriction/dilation of the pupil (see Figure 1 and section “Pupil and Iris”), serves to dark- or light-adapt the eye. Pigment migration does not seem to be uniformly fast throughout the entire retina; in the photoreceptors within the central stripe, which have less pigment than the cells in other retinal regions (Young, 1962b), pigment migration is slower during light adaptation, but faster during dark adaptation than in the remainder of the retina (Hess, 1905; Young, 1963).

In *Octopus fangsiao (O. ocellatus)*, dopaminergic efferents from the optic lobe seem to cause screening pigment migration during the dark adaptation process (Gleadall et al., 1993). In *O. vulgaris*, this has yet to be studied.

The photoreceptors of the octopus retina narrow before passing the basement membrane that separates their distal parts from the proximal segments that carry the cell nuclei (Figure 3). Finally, the photoreceptors give rise to axons. Within this region, called the retinal plexus, two types of interactions can be found: (1) interactions between photoreceptors, mediated by fine collateral fibers branching from the proximal part of the photoreceptors, and (2) interactions between centrifugal cells, which are efferents from the plexiform zone of the optic lobes, and photoreceptors (Young, 1962b; Boycott et al., 1965; Tonosaki, 1965; Lund, 1966; Patterson and Silver, 1983). In the central stripe of the retina, the proximal segments of the photoreceptors are longer, and the retinal plexus is thicker than in the rest of the retina (Young, 1963). Three studies have described synapses and transmitters in the retina of *Octopus vulgaris,* among other species (Gray, 1970; Lam et al., 1974; Silver et al., 1983). There is accumulating evidence that the photoreceptors are cholinergic, whereas the centrifugal cells are dopaminergic.

The axons of the photoreceptors leave the eye in bundles of approximately 20 axons, each through holes in the sclera (Patterson and Silver, 1983). Before these axon bundles enter the optic lobe, the bundles decussate: the dorsal retina projects to the ventral optic lobe and vice versa (Boycott et al., 1965; Lettvin and Maturana, 1965; Patterson and Silver, 1983). The optic lobes are composed of the cortex and a central medulla, and most photoreceptors axons terminate in the outer plexiform zone of the cortex of the optic lobe (Young, 1960, 1962a, 1971; Dilly et al., 1963).

### Dichroism of the Retina and Polarization Sensitivity

The rhabdomeres of the photoreceptors are arranged either horizontally or vertically. Each rhabdomere consists of densely packed straight microvilli that, because of the regular arrangement of the rhabdomeres, are oriented perpendicular to each other (Wolken, 1958; Young, 1960, 1971). With the alignment of the visual pigment with the long axis of the tubules (Roberts et al., 2011), each rhabdomere is a dichroic analyzer that absorbs light polarized parallel to the tubules maximally. This regular retinal arrangement is thus most likely the basis for the ability of octopus to perceive polarized light (Moody and Parriss, 1960, 1961; Rowell and Wells, 1961; Lettvin and Pitts, 1962; Moody, 1962; Tasaki and Karita, 1966; Sugawara et al., 1971; Shashar and Cronin, 1996).

Numerous functions of polarization sensitivity have already been described for cephalopods in general, including object detection or recognition, communication or navigation, among other (for review see Mäthger et al., 2009; Shashar, 2014). However, it still remains to be determined what role polarization sensitivity plays in *Octopus vulgaris* in particular, as most evidence in this respect has been collected in other cephalopod species, so far. Additionally, it remains to be determined whether octopus possesses true polarization vision as proposed by Shashar and Cronin, 1996, a view that has been challenged by Nilsson and Warrant (1999).

### Photoreceptor Density, Spatial, and Temporal Resolution

Given an eye size of approximately 2 cm (see section “Eye Size and Ocular Dimensions”), the octopus retina covers an area of 1–4 cm^2^ (Wolken, 1958; Young, 1963). In this retina, 2–3 × 10^7^ photoreceptors cells are found with a cell density varying between 18,000–22,000 cells/mm^2^ in the periphery and approximately 55,000 cells/mm^2^ in the central stripe (Young, 1960, 1962b, 1963, 1971). In the central stripe, the rhabdoms are longer and thinner than in the periphery; rhabdom diameters as small as 4 μm have been found in the stripe, while rhabdoms in the periphery had diameters of up to 10 μm (Young, 1963). The higher rhabdom density in the central retinal stripe is strongly indicative of higher spatial resolution in this area, even though this has not been measured directly with electrophysiological methods; for electrophysiological studies in octopus, the reader is referred to previous studies (Tasaki et al., 1963a,b; Boycott et al., 1965; Lettvin and Maturana, 1965; Hamasaki, 1968a,b; Tsukahara et al., 1973). In accordance with the foregoing, a horizontal area of increased spatial resolution would be highly adaptive in bottom-living animals (Muntz, 1977; Talbot and Marshall, 2010).

Visual acuity was assessed with two different behavioral approaches, in a discrimination experiment using gratings (Sutherland, 1963b) as well as in an optomotor study (Packard, 1969). The first approach assessed visual acuity as 1.7 cycles/degrees or better for animals weighing 250–500 g. The second assessed visual acuity as 0.6–1.1 cycles/degrees for two groups of very small animals with average weights of 0.27 g and 2.7 g, and 1.1 cycles/degrees or better for animals weighing 17 g; all values are estimates based on the assumption that the animals were in the center of the optokinetic drum. Due to several aspects related to the experimental procedure, these studies may have underestimated the visual acuity of octopus. This possibility is supported by another discrimination experiment on grating visual acuity in *Octopus pallidus* and *Octopus australis* whose visual acuity was assessed as 3.1–6.8 cycles/degrees (Muntz and Gwyther, 1988). Considering this acuity range, the octopus visual acuity would be comparable to the visual acuity of cats or fowls (Rahmann, 1967). Generally, the visual acuity of octopus might vary with illumination, as the receptive field of single receptors will probably be smaller when the pigment has migrated to the distal tip of the receptors in bright light (Lettvin and Pitts, 1962; Sutherland and Carr, 1963; Young, 1963); an aspect that still needs to be fully worked out in octopus.

The temporal resolution of the eye, as measured by flicker fusion frequency, has been determined in both *Octopus vulgaris* and *Octopus briareus* as 72 Hz with a stimulus intensity of 4.5 × 10^6^ cd/m^2^ by Hamasaki (1968b). The flicker fusion frequency decreases relatively fast reaching only 20 Hz when stimulus intensity was decreased by 4 logarithmic units. As the values given are averages from the two species of octopus, it would be interesting to document the flicker fusion frequency for *Octopus vulgaris* in particular.

### Visual Pigment and Color Vision

*Octopus vulgaris* possesses only one visual pigment within its photoreceptors, an R-type-opsin (Cronin and Porter, 2014) which absorbs maximally at 475 nm with a β-band at 360 nm (Brown and Brown, 1958; Kropf et al., 1959; Hamasaki, 1968a). Generally, the visual pigments of octopods seem to be less well matched to the light environment than the pigments of squids and cuttlefish. It is speculated that a fine-tuning of the pigments might not be under selective pressure in octopods in contrast to squids and cuttlefish as other senses such as haptics or chemoreception might be more important than vision in these benthic animals (Chung and Marshall, 2016).

In line with the presence of only one visual pigment, most studies have concluded that *Octopus vulgaris* is color-blind (Piéron, 1914; Bierens de Haan, 1926; Messenger et al., 1973; Messenger, 1977; Kawamura et al., 2001), though the work of Fröhlich, Goldsmith, and Kühn suggest otherwise (Fröhlich, 1914a,b; Goldsmith, 1917a,b; Kühn, 1950). However, in these old color vision studies, either experiments were not adequately controlled for the brightness of the stimuli or stimuli were adjusted in brightness on the basis of a human brightness discrimination ability that likely differs from the brightness discrimination ability of octopus. Moreover, these studies were not designed to examine a color vision mechanism recently simulated for *Octopus australis* by Stubbs and Stubbs (2016a). This color vision mechanism exploits the longitudinal chromatic aberration of the lens; thus, even monochromats should be able to obtain color information this way. Although this mechanisms has been questioned (Gagnon et al., 2016; Stubbs and Stubbs, 2016b), it would be interesting to test it in the context of the mystery of color-blind camouflage and the question of what role the eyes and/or photoreceptors in the skin (Ramirez and Oakley, 2015) play in background matching by cephalopods generally. Stubbs and Stubbs speculate that this mechanism might also help to explain why some cephalopods have developed colorful intra-specific signals (Stubbs and Stubbs, 2016b).

To date, the only cephalopod known to possess more than one pigment, the classic precondition for color vision, is *Watasenia scintillans*; it has three visual pigments based on vitamin A1 (λ_max_ = 484 nm), vitamin A2 (λ_max_ = 500 nm), and 4-hydroxyretinal (λ_max_ = 470 nm) (Matsui et al., 1988a,b; Seidou et al., 1990; Kito et al., 1992; Michinomae et al., 1994). A putative color vision faculty in the firefly squid is supported by the existence of a banked retina that compensates for this animal’s lens not being corrected for longitudinal chromatic aberration (Kröger and Gislen, 2004).

## Discussion

This review demonstrates that several aspects of vision of *Octopus vulgaris* have been investigated in some detail. Nevertheless, large gaps remain in our understanding of vision for this species, notwithstanding the fact that the common octopus has been an object of scientific study for more than 150 years. In our opinion, one of the largest gaps in our knowledge stems from the poor understanding of the dioptric apparatus of octopus. In addition, the primary functions of vision — including visual acuity, brightness discrimination, depth perception, motion detection, polarization and color vision — have not been conclusively investigated, and thus some enduring mysteries (in particular, color-blind camouflage) persist to the present day.

Taken together, the current array of published studies on the eye of *Octopus vulgaris* — many of which are reviewed here — helps us to understand adaptations of the visual system to lifestyle and habitat. To provide some examples, characteristics of the visual system of octopus such as specifics of the pupil or the retina mirror the benthic lifestyle of adult octopus which can even inhabit shallow water: an environment in which it experiences high light intensities from above and drastic light changes when leaving its den during the day. Moreover, the large eye movements and aspects that camouflage the animal or the eye specifically reinforce the fact that octopus is a soft-bodied animal that falls prey to many animals. Future studies will allow completion of a picture of vision in *Octopus vulgaris*. Detailed insight will thus be obtained regarding the world of a fascinating invertebrate which otherwise spends its life in a habitat that is still not easily accessible to humans.

## Author Contributions

FH wrote the first version of the manuscript. AK contributed comments and suggestions. Both authors approved the final version of the manuscript.

### Conflict of Interest

The authors declare that the research was conducted in the absence of any commercial or financial relationships that could be construed as a potential conflict of interest.
